# Infective Endocarditis: An Embolic Case

**DOI:** 10.7759/cureus.27489

**Published:** 2022-07-30

**Authors:** Ana Amorim, Ana Santos, Sara Trevas

**Affiliations:** 1 Anesthesiology, Hospital Central do Funchal, Funchal, PRT; 2 Internal Medicine Department, Hospital São Francisco Xavier, Lisboa, PRT

**Keywords:** acute cardiac care, trans-esophageal echocardiogram, echo cardiogram, septic embolic stroke, infective endoc

## Abstract

Infective endocarditis is a sudden illness that rapidly causes cardiac and extracardiac injury. Embolic material travels into the arterial circulation causing embolic events in 20-50% of patients. The brain is one of the most frequent sites of embolism that potentially interferes with treatment options. Neurologic complications are the presenting symptom in 20% of the cases being associated with poor prognosis (45% of deaths versus 24% in patients without these complications).

This is the case of a 63-year-old male patient presenting with main clinic of stroke. Multifocal signs and past aortic valvuloplasty raised the suspicion of infective endocarditis and antimicrobial therapy was initiated despite an initial negative transthoracic echocardiography (TTE). Imaging study revealed vascular lesions in different arterial territories of the brain, some of them with hemorrhagic transformation and multiple splenic and renal areas of infarction. Hemodynamic instability and acute pulmonary edema developed just before surgery. Transoesophageal echocardiography (TEE) confirmed a typical image of vegetation, conditioning severe aortic regurgitation, and a perivalvar abscess with fistulization to the right ventricle. Both were surgically repaired. The immediate postoperative period was characterized by cardiogenic shock, but the patient evolved favorably being transferred to the hospital ward where he continued his motor recovery.

Early surgery is a mainstay in the treatment of infective endocarditis, reducing the embolic risk. Once happened, neurologic embolization may worsen the prognosis and raise doubts about further deterioration or hemorrhagic conversion following cardiopulmonary bypass. Optimal time interval between ischemic stroke and surgery has not yet been determined but recent data favour early surgery that, when indicated, should not be delayed.

Most of the embolic events occur before admission making presentation variable. Clinical suspicion is highly important to the prompt institution of antibiotic therapy and the avoidance of subsequent embolic events. TTE is a sensitive tool in the diagnosis of endocarditis, but a negative result does not exclude the diagnosis specially when endocarditis is clinicalliy expected. Imaging should be systematically performed in the course of the disease to detect new and relevant complications, always being aware of the higher sensitivity of TEE to detect intracardiac complications.

## Introduction

Infective endocarditis (IE) is a sudden illness that rapidly causes cardiac and extracardiac injury when embolic material travels into the arterial circulation. With an incidence from four to seven cases per 100,000 population [1] in developed countries, untreated it progresses to death. Medical history is variable depending on the causative agent, pre-existing heart disease and embolic events which occur in 20-50% of patients. 

The brain is one of the most frequent sites of embolism, mainly in left-sided endocarditis. Neurologic complications are the presenting symptom in 20% of the cases, interfering with treatment options, and being associated with poor prognosis (45% of deaths versus 24% in patients without these complications) [2,3]. Modified Duke criteria, which include intracranial hemorrhage as a minor criterion, are useful, but clinical judgment is necessary.

This is the case of a 63-year-old male patient presenting with main clinic of stroke whose multifocal signs and past aortic valvuloplasty raised the suspicion of infective endocarditis. Antimicrobial therapy was initiated despite an initial negative transthoracic echocardiography (TTE).

## Case presentation

**Figure 1:**
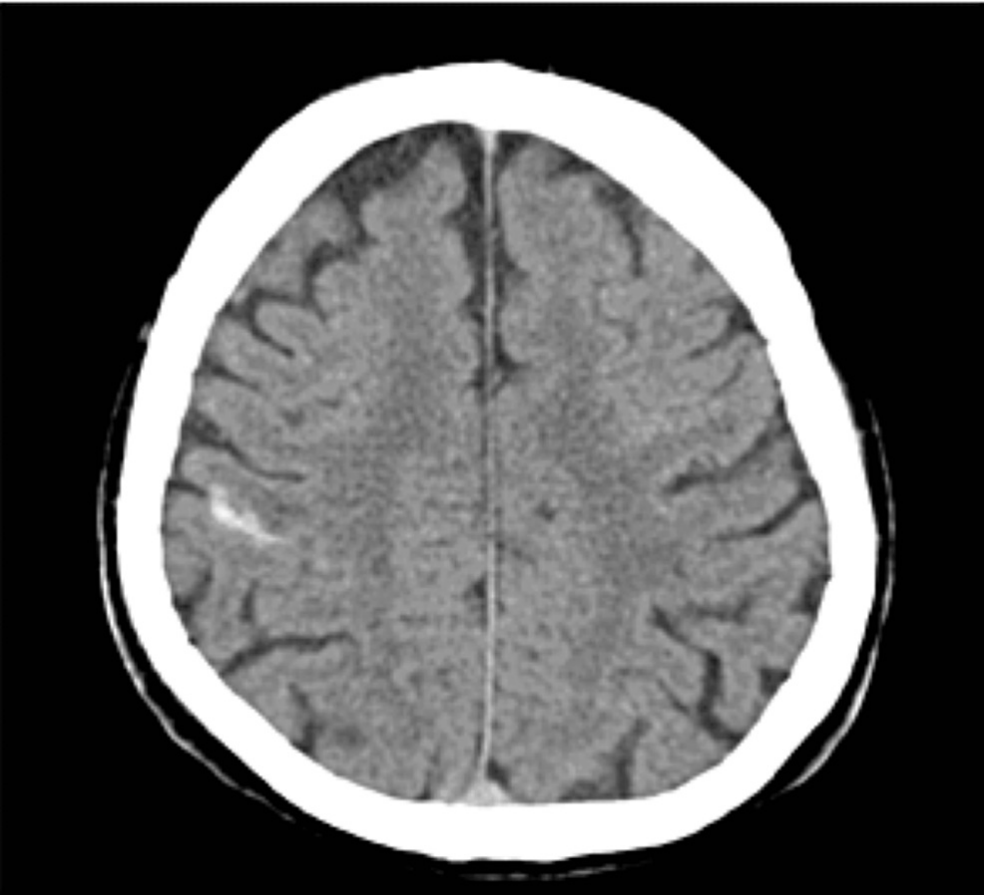
Brain CT scan Head CT scan showing a focal hemorrhage filling the right postcentral sulcus associated with hypodensity in the postcentral gyrus corresponding to ischemia or edema.

A 63-year-old man was admitted to a secondary hospital with a sudden left-side hemiparesis, left leg hypoesthesia and right leg weakness, accompanied by fever, vomiting and diaphoresis. With medical history of aortic valvuloplasty 15 years ago the patient denied subsequent complications, symptoms of congestive heart failure (HF) or angina. On admission, he presented with regular pulse of 94 bpm, no murmurs, blood pressure of 114/75 mmHg, normal breath sounds, but was polypneic with respiratory alkalosis and decreased arterial oxygenation (partial pressure of oxygen [PaO2] of 63 mmHg on room air). Laboratory tests revealed a white cell count of 15.300/mL (86% polymorphonuclear cells), high c-reactive protein (CRP) level of 353 mg/dl, 77,000/mL platelets, serum creatinine level of 1.38 mg/dl and urinalysis suggestive of infection. Head CT scan showed a focal hemorrhage filling the right postcentral sulcus associated with hypodensity in the postcentral gyrus corresponding to ischemia or edema. MRI scan was performed revealing multiple punctiform brain lesions in different vascular territories indicating an embolic source, some of them with haemorrhagic transformation (Figure 1). Blood cultures were positive for methicillin-sensitive S. aureus (MSSA). Despite TTE not revealing any structure indicative of vegetation, antimicrobial therapy with flucloxacillin 12g per day was started, assuming the frequent association between MSSA bacteraemia and endocarditis. 

**Figure 2:**
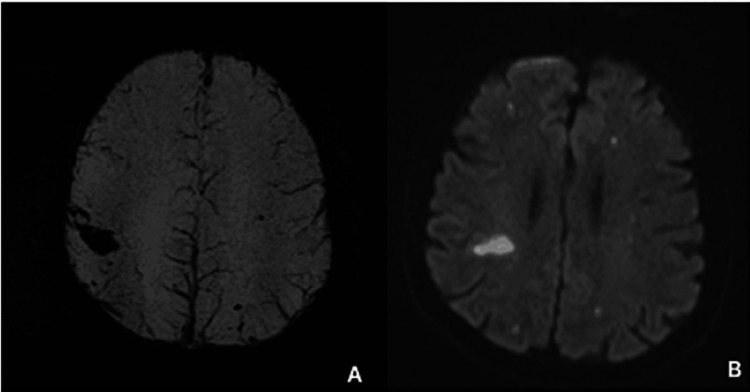
Brain MRI scan Brain MRI showing blooming foci in susceptibility weighted imaging (SWI) (A) corresponding to regions with restriction in the diffusion study (B) and assumed as ischemic injuries with discrete foci of hemorrhagic transformation. Multiplicity and distribution suggesting an embolic ethology.

In the meanwhile, vascular phenomena were noticed with Janeway lesions in fingers of both hands and plantar surface of the feet. At that time, the patient presented one major criterion according to Duke´s criteria (two blood cultures positive for MSSA) and three minor criteria including vascular phenomena, predisposing cardiac condition and a fever that persisted until the seventh day of antibiotics, despite prompt decrease in CRP. An abdominal CT scan was performed revealing multiple splenic infarcts, the largest with 9cm, as well as areas of renal infarction the largest with 7cm.

Echocardiography was repeated 16 days after the admission using transoesophageal approach. It revealed severe aortic regurgitation, a typical image of vegetation next to the anulus and a perivalvar abscess with fistulization to the right ventricle. CT scan of the brain was repeated demonstrating the already established small ischemic lesions and a new one, in the left front area, without new signs of intracranial haemorrhage. At the time of arrival to our hospital, and 20 days after the initial admission, the patient had already developed hemodynamic instability and acute pulmonary edema. Despite the infection starting to subside and the fever disappearing, heart failure and intracardiac abscess fistulae were, at that point, a major indication for urgent cardiac surgery. The perivalvar abscess and fistulization were repaired and aortic valve replaced using biological prosthesis. The immediate postoperative period was characterized by cardiogenic shock with need for aminergic and vasopressor support as well as renal failure with no need for renal replacement therapy. Despite this, the hospitalization evolved favourably, the patient stayed in our ICU for 10 days before being transferred to the hospital ward where he continued his motor recovery after the stroke.

## Discussion

Early surgery is a mainstay in the treatment of infective endocarditis particularly in the presence of progressive HF and uncontrolled infection. Severe left valve regurgitation may lead to HF, the most common indication for surgery and present in 42-60% of native valve endocarditis [2]. 

Intracardiac complications can also be the result of uncontrolled infection, whose definition is not entirely established. Arbitrarily defined as persisting positive cultures and fever after seven to 10 days of antibiotic treatment, it can cause locally progressive infection with abscess, pseudoaneurysms and fistulae requiring prompt intervention. Embolic complications are a frequent reason for persistent fever and local infection since the risk of embolization increases with vegetation size. Silent cerebral embolisms occur in up to 60% of cases with neurologic symptoms in 15-30% of patients [2]. 

Once it has happened, neurologic embolization may worsen the prognosis and raise doubts about further neurologic deterioration or hemorrhagic conversion following cardiopulmonary bypass. The optimal time interval between ischemic stroke and surgery has not yet been determined but, when indicated, should not be delayed. Still, if intracranial hemorrhage is present, neurological decline is expected in about 33.3% of the cases and surgery should generally be postponed for one month or more [4,5]. The decision should be individualized, but recent data favour prompt surgery even in presence of intracranial hemorrhage [2]. In this clinical case, the neurologic damage of the patient was not severe, and the hemorrhage was limited to small hypointense foci, with no progression in the subsequent evaluation prior to surgery. The surgery was lifesaving in the face of heart failure and was performed 20 days after the initial stroke. No further neurological deterioration occurred. 

## Conclusions

Most embolic events occur before admission, making presentation variable. Clinical suspicion is highly important in the prompt institution of antibiotic therapy and avoidance of subsequent embolic events.

TTE is a sensitive and specific tool in the diagnosis of endocarditis, but a negative result does not exclude the diagnosis, especially when endocarditis is expected. Imaging should be systematically performed in the course of the disease to detect new and relevant complications always being aware of the higher sensitivity of TEE to detect intracardiac complications.
